# Impact of traffic congestion on spatial access to healthcare services in Nairobi

**DOI:** 10.3389/frhs.2022.788173

**Published:** 2022-11-16

**Authors:** Nyamai Mutono, Jim A. Wright, Mumbua Mutunga, Henry Mutembei, S. M. Thumbi

**Affiliations:** ^1^Wangari Maathai Institute for Peace and Environmental Studies, University of Nairobi, Nairobi, Kenya; ^2^Center for Epidemiological Modelling and Analysis, University of Nairobi, Nairobi, Kenya; ^3^Paul G. Allen School for Global Health, Washington State University, Pullman, WA, United States; ^4^School of Geography and Environment Science, University of Southampton, Southampton, United Kingdom; ^5^Institute of Tropical and Infectious Diseases, University of Nairobi, Nairobi, Kenya; ^6^Department of Clinical Studies, University of Nairobi, Nairobi, Kenya; ^7^Institute of Immunology and Infection Research, School of Biological Sciences, University of Edinburgh, Edinburgh, United Kingdom

**Keywords:** traffic congestion, healthcare accessibility, universal healthcare access, sub-Saharan Africa, catchment model

## Abstract

**Background:**

Geographic accessibility is an important determinant of healthcare utilization and is critical for achievement of universal health coverage. Despite the high disease burden and severe traffic congestion in many African cities, few studies have assessed how traffic congestion impacts geographical access to healthcare facilities and to health professionals in these settings. In this study, we assessed the impact of traffic congestion on access to healthcare facilities, and to the healthcare professionals across the healthcare facilities.

**Methods:**

Using data on health facilities obtained from the Ministry of Health in Kenya, we mapped 944 primary, 94 secondary and four tertiary healthcare facilities in Nairobi County. We then used traffic probe data to identify areas within a 15-, 30- and 45-min drive from each health facility during peak and off-peak hours and calculated the proportion of the population with access to healthcare in the County. We employed a 2-step floating catchment area model to calculate the ratio of healthcare and healthcare professionals to population during these times.

**Results:**

During peak hours, <70% of Nairobi's 4.1 million population was within a 30-min drive from a health facility. This increased to >75% during off-peak hours. In 45 min, the majority of the population had an accessibility index of one health facility accessible to more than 100 people (<0.01) for primary health care facilities, one to 10,000 people for secondary facilities, and two health facilities per 100,000 people for tertiary health facilities. Of people with access to health facilities, a sub-optimal ratio of <4.45 healthcare professionals per 1,000 people was observed in facilities offering primary and secondary healthcare during peak and off-peak hours.

**Conclusion:**

Our study shows access to healthcare being negatively impacted by traffic congestion, highlighting the need for multisectoral collaborations between urban planners, health sector and policymakers to optimize health access for the city residents. Additionally, growing availability of traffic probe data in African cities should enable similar analysis and understanding of healthcare access for city residents in other countries on the continent.

## Introduction

An estimated 3.6 million deaths in low- and middle-income countries (LMICs) are associated with non-utilization of healthcare (1). Timely healthcare access encompassing physical access to quality care, acceptability and financial access remains low in many developing economies despite its potential to substantially reduce mortality (2, 3).

Health is one of the measures of quality of life, and access to health facilities and to a skilled workforce in facilities has been prioritized by the United Nations through the Sustainable Development Goals. Goal 3 addresses better access to healthcare and the healthcare workforce with Target 3.8 seeking to achieve universal health coverage (UHC) for all by 2030, while Target 3.c aims to recruit, train, and retain the healthcare workforce in developing countries (4). Population access to these resources has been evaluated using geographical accessibility (3, 5).

Geographical accessibility through distance or travel time has been reported as a significant barrier to effective treatment (6, 7). Optimal placement of health facilities that minimizes travel distance to health facilities while maximizing population coverage has been advocated (8, 9). Research focusing on LMICs has used cost surfaces (for example, the World Health Organization's (WHO) Access Mod software) and the two-step floating catchment area (2SFCA) method to measure geographical access (3, 7, 10). However, cost surfaces are less well-suited for quantifying patient travel in cities experiencing congestion as they expand and the associated challenges of timely access to urban secondary healthcare. On the other hand, the 2SFCA method calculates geographical accessibility of healthcare services through use of the gravity model to analyse the ratio of healthcare supply and population demand, while often incorporating patient mode of transport and road networks, and accounting for the population that has more than one health facility in its proximity (10, 11). However, there is limited research on the use of this method to assess the effect of traffic on the proportion of population with access to a health facility and the healthcare workforce, particularly in cities in LMICs which are characterized by rapid urbanization and under-developed road infrastructure (12). Similarly, the healthcare workforce in Sub Saharan Africa is considerably under-resourced, with Kenya facing an unequal distribution of these personnel in the existing health facilities (13, 14).

Studies of peak and off-peak travel time to health facilities in Africa have primarily used methods that employ road networks (3, 15). However, these studies focus on geographic access to emergency care without incorporating the ratio of population to healthcare workforce. Additionally, these studies consider traffic conditions for broad categories of road types, without including disaggregated traffic data. We investigated geographical access to healthcare facilities and professionals in an African city using the 2SFCA method while incorporating traffic data to address two research questions: (i) How are healthcare facility catchment areas affected by traffic congestion? (ii) How does traffic congestion influence the distribution of healthcare professionals to population across healthcare facilities?

## Materials and methods

### Study area

This study was conducted in Nairobi County, the most populous city in Kenya. The 2019 census data estimated Nairobi's population at 4.4 million with a population density of 6,247 per km^2^ (16).

**Figure 1:**
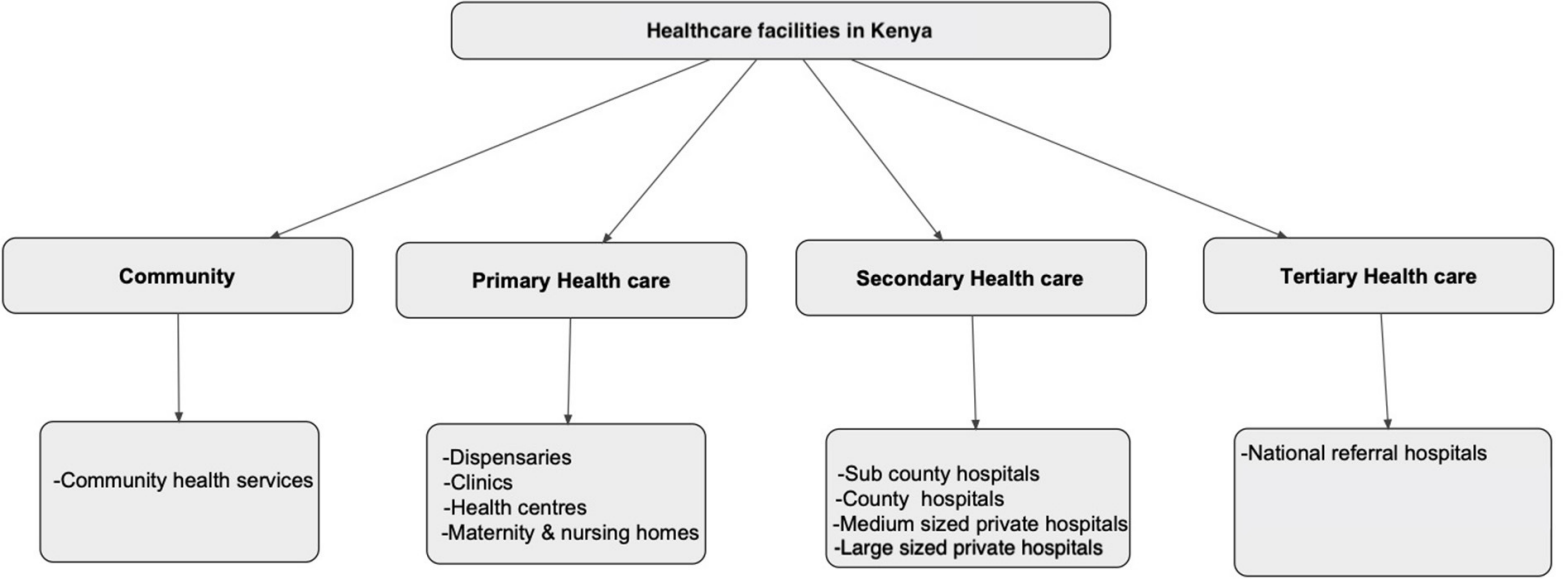
The four categories of healthcare in Kenya. Source: Kenya Health Policy 2014–2030 (17).

In Kenya, health facilities are divided into four tiers based on the level of care offered. Tier I comprises community health services, providing mainly community health volunteer visits and health promotion messages. Tier II comprises the lowest level of health facilities that offer primary healthcare services (dispensaries, clinics, health centers and maternity centers). Tier III comprises sub-County hospitals, medium-sized and large private hospitals, County hospitals and large private teaching hospitals, which offer secondary healthcare services while, Tier IV are national referral hospitals that offer tertiary healthcare services (Figure 1) (17). Tiers I and II focus on health promotion and prevention, while Tiers III and IV prioritize curative services (17).

**Figure 2:**
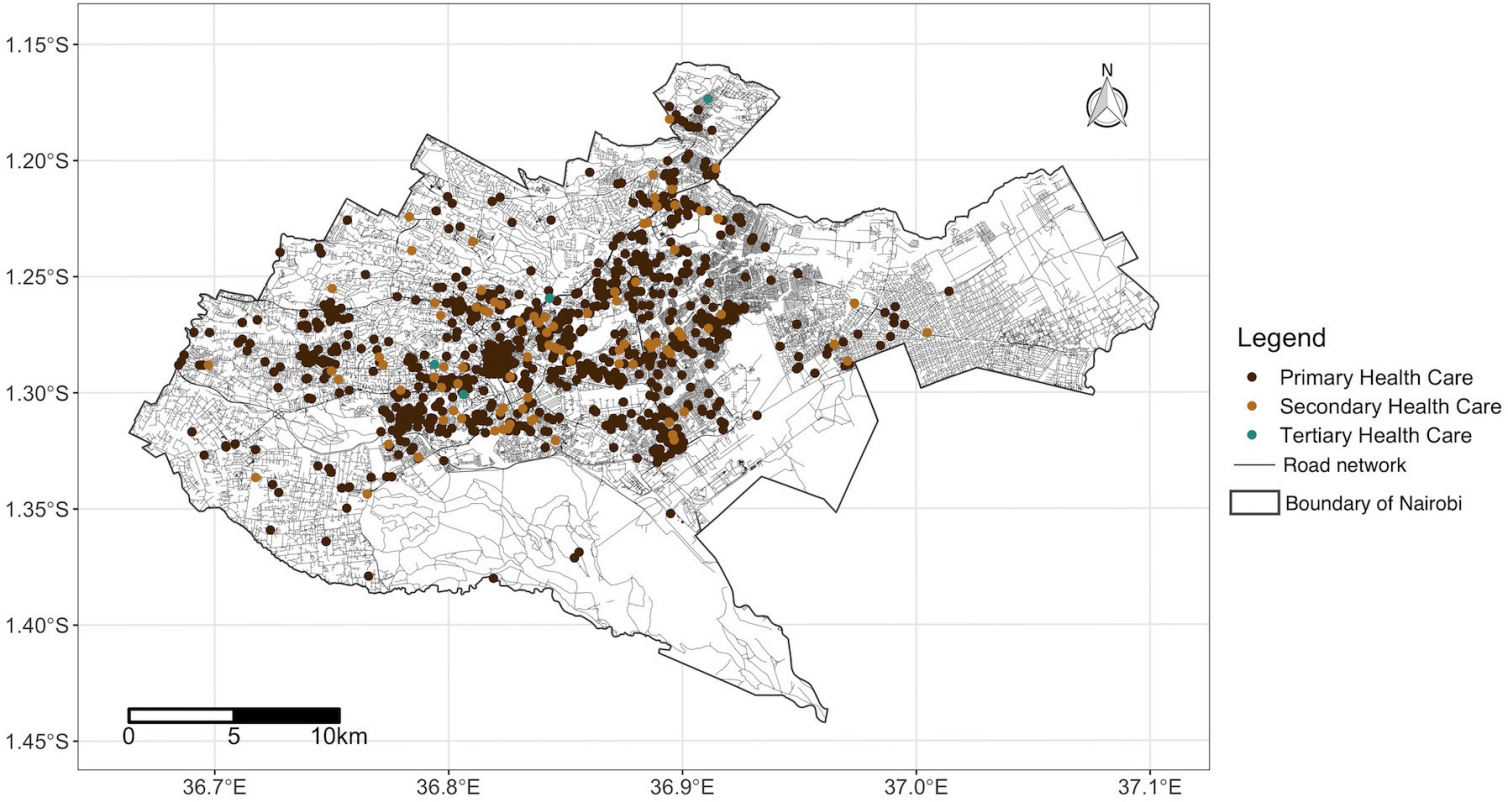
Road network coverage and locations of health facilities in Nairobi County, Kenya source: OpenStreetMap, Ministry of Health. Shapefile source: GADM (18).

Since 2013, delivery of health services has been a devolved function of the county government, with the national government retaining the mandate of policy formulation and regulation (19). In Nairobi County, service delivery *via* public health facilities is under the auspices of the Nairobi Metropolitan Services (NMS) (20). In 2020, Nairobi County had 1,042 health facilities registered under the Ministry of Health (21), yielding a ratio of 23.7 health facilities per 100,000 people. The majority (91%) of these facilities offered primary healthcare services while ~9 and ~1% offered secondary and tertiary healthcare services, respectively. In addition, 74% of these health facilities were privately owned (Figure 2).

Kenya is one of two African countries for which the Environmental Systems Research Institute (ESRI) have online historical traffic data available to support drive-time calculations (22). Approximately 88% of motorized public transport trips by urban residents in Kenya are in a *matatu* (a type of minibus) (23).

### Facility geocodes

In September 2020, we downloaded a list of health facilities registered in Nairobi from the Ministry of Health—Kenya Master Health Facility List (21). Public health facility coordinates were extracted from a previous study (24). Geocodes of private health facilities were obtained by use of street level precision of Google Earth Pro's geocoding service (version 7.3) which has low positional error (25).

### Population data

We used the 2019 population data from WorldPop which are calculated using official country census data, administrative boundary maps and ancillary geospatial data sets of road networks, hospital facilities, satellite imagery and building or settlement maps (26). The WorldPop group projected aggregate population counts from the 2009 census for sub-locations (areas with a mean population of 5,400) to 2019 and re-distributed these within each boundary onto constituent 100 by 100 meters resolution grid cells. Using a random forest dasymetric mapping algorithm, which is an ensemble non-parametric modeling approach, Worldpop predicted the population in every grid cell from census data and a weighting scheme from covariates such as distance to urban areas, unvegetated areas, settlement built-up areas, water bodies, roads and night-time lights, among others (27).

### Health facility catchment area

To estimate the catchment areas for healthcare facilities, we used ESRI routing services which collect historical traffic data from probes, which are global positioning system (GPS) enabled devices embedded in vehicles or carried by passengers for example cell phones (28). The probes are able to relay their location in real time at frequent refresh rates. This system calculates distance- and time-based catchment areas, accounting for mode of transport, road networks and traffic regulations (29). To analyse the impact of traffic congestion on motorized access to healthcare services relative to the catchment areas, we selected three travel cut-off times (15, 30, and 45 min) and compared travel times during randomly selected peak- and off-peak traffic hours. Peak hours included Monday 8 am and Wednesday 6 pm, while off-peak hours included Sunday 8 am and Saturday 6 pm.

We computed the ratio of health facilities to the population using a 2SFCA method adapted from Ahmed et al. (30). In this method, the catchment areas for each health facility within a given threshold are calculated and the population within the catchment area tabulated. For population falling within the catchment area of more than one health facility per category, the ratios are summed up to assess the accessibility index for each population.

(i) Ratio (R) of health facilities (F) to population (P)

The population (P) that was within the travel time (*tt*_0_) of each health facility (F) was calculated and the ratio of health facilities to the population *R*_*f*_ was computed using:


$$R_{f}=\frac{F}{\sum_{f\in tt_{f\leq tt_{0}}}P_{f}}$$

We then calculated the population accessibility index by computing the ratio of health facilities to population in 100 by 100 meter grid cells. For populations with access to >1 healthcare facility within the travel threshold of each point representing the population, the ratio of healthcare facility to population for each level of facility was summed up. Population that was not within the catchment area of at least one health facility per category was defined as underserved. We used the *Z*-test to compare facility-level populations accessing health facilities during peak and off-peak hours.

### Ratio of healthcare professionals to population

We used average staffing levels for healthcare professionals across healthcare categories as reported in the Kenya Service Provision Assessment (KSPA) Survey of 2010 (Table 1) (31). The KSPA survey included 703 facilities which were randomly selected from the Master Health Facility List. Data on staffing were collected and averaged according to the level of care.

**Table 1:** The average number of healthcare professionals per facility in Kenya in 2011, by healthcare category.

**Health facility category**	**Primary**	**Secondary**	**Tertiary**
Nurses/midwives	8	13	288
Clinical officers	3	4	30
Doctors	-	2	10
Total	11	19	328

National Coordinating Agency for Population and Development et al. (31).

(ii) Ratio (R) of healthcare professionals (HP) to population (P)

The population (P) that was within the travel time (*tt*_0_) of healthcare professionals (HP) in each facility (*f* ) was calculated and the ratio of healthcare professionals to the population *R*_*hp*_ was computed using:


$$R_{hp}=\frac{HP}{\sum_{f\in tt_{f\leq tt_{0}}}P_{hp}}$$

We then calculated the population accessibility index by computing the ratio of healthcare professionals to population in 100 by 100 meter grid cells. For populations with access to >1 health facility within the travel threshold of each point representing the population, the ratio of healthcare professionals to population for each facility was summed. Populations outside facility catchment areas were considered inaccessible to healthcare professionals. We used the WHO recommendations of 4.45 healthcare professionals (doctors, clinical officers, nurses, midwives) per 1,000 people to classify access as sub-optimal (<4.45:1,000) or optimal (>4.45:1,000) (32). To cater for edge effects, we applied a 2.5-kilometer buffer from administrative boundaries, recalculated the healthcare professional to population ratio and the accessibility index, and used the new values to classify access as either optimal or sub-optimal. The analysis and visualization was conducted using R statistical software version 4.0.4 (33).

## Results

To quantify geographic access to healthcare, we first present data concerning the proportion of population within 15-, 30- and 45-min drive of the nearest primary, secondary and tertiary healthcare facility, examining how these proportions vary during peak vs. off-peak travel times. We then present population to healthcare professional ratios, drawing on the related two-step floating catchment analysis.

### Catchment areas for health facilities

The proportion of the population within the catchment areas of each level of healthcare that could access the healthcare facilities within ≤ 15, 30 and 45 min during off-peak hours was significantly reduced during peak traffic times. This was observed for all three levels, except for secondary health facilities which were accessible to the whole population during peak and off-peak hours at 45 min threshold (Table 2). During peak hours, 45, 20, and 17% of the population could access primary, secondary, and tertiary health facilities, respectively, within 15 min. During off-peak hours, access to primary, secondary, and tertiary health facilities increased by 3, 23, and 13% (*p* < 0.001) respectively. At 30 min, >50% of the population was able to access the three levels of health facilities, with the greatest proportion having access to primary healthcare during peak (68%) and off-peak (75%) hours. Tertiary healthcare had the lowest population access (55%) at 30 min during peak hours.

**Table 2:** Distribution of Nairobi's 4.1 million residents within 15, 30- and 45-min drive-time to different levels of health facilities during peak and off-peak traffic hours.

**Healthcare**	**Time**	**Population in**		***P*-value**
**level**	**(min)**	**millions (%)**		
		**Peak hours**	**Off-peak hours**	
Primary	≤ 15 min	1.8 (45%)	2.0 (48%)	<0.001
	≤ 30 min	2.8 (68%)	3.1 (75%)	<0.001
	≤ 45 min	3.9 (95%)	4.0 (97%)	<0.001
Secondary	≤ 15 min	0.8 (20%)	1.8 (43%)	<0.001
	≤ 30 min	2.5 (62%)	2.9 (71%)	<0.001
	≤ 45 min	4.1 (100%)	4.1 (100%)	1
Tertiary	≤ 15 min	0.7 (17%)	1.3 (30%)	<0.001
	≤ 30 min	2.3 (55%)	2.9 (71%)	<0.001
	≤ 45 min	3.6 (86%)	3.7 (91%)	<0.001

### Geographical access to healthcare

**Figure 3:**
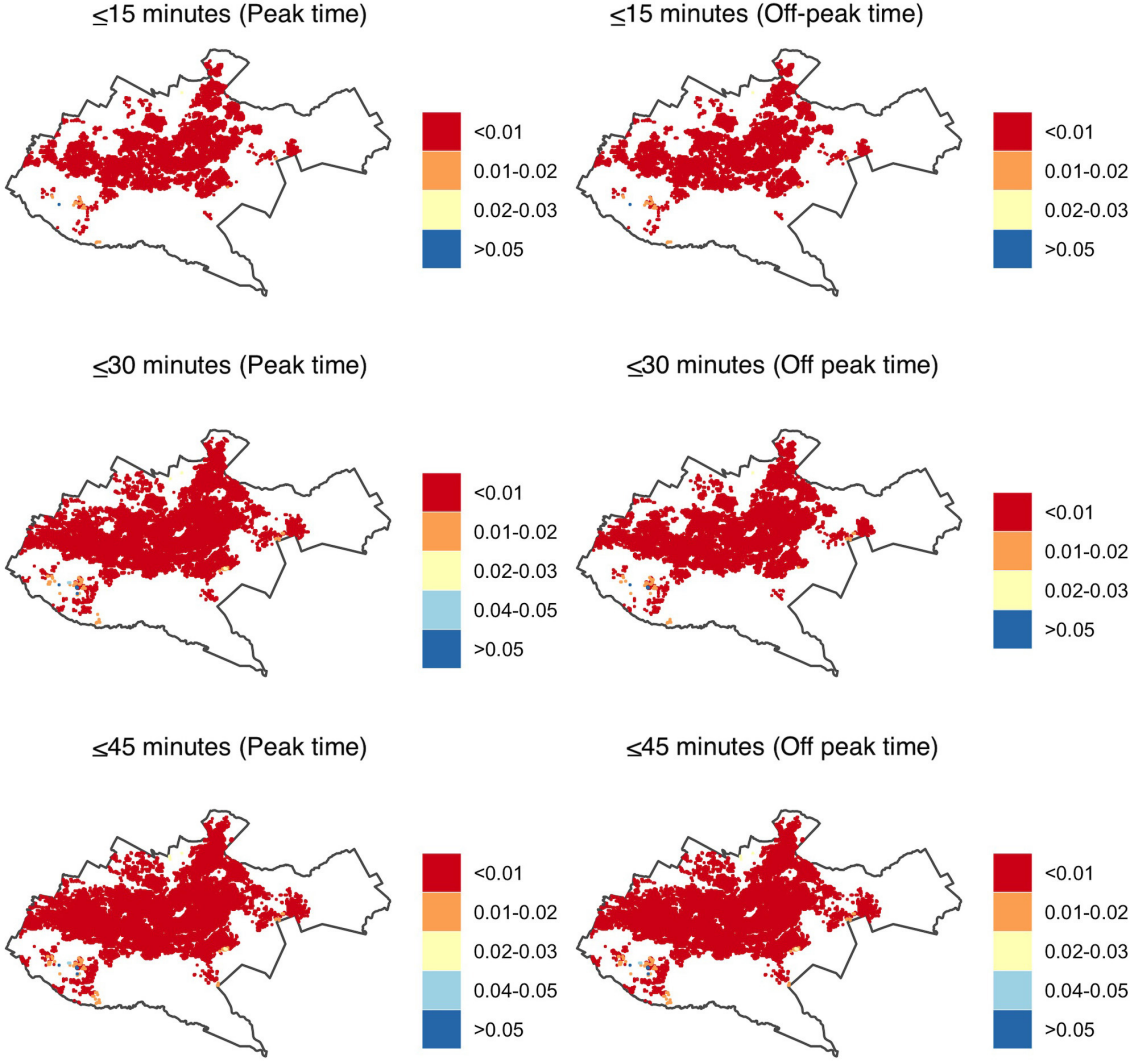
Accessibility index (population: facility ratios) for primary health care in Nairobi within 15-, 30- and 45-min drive during peak and off-peak traffic times. The white areas have no accessibility to health facilities. Shapefile source: GADM (18).

The spatial coverage of facilities offering primary healthcare was predominantly in the central region of Nairobi County during both peak and off-peak hours, with the peripheries underserved both during the peak and off-peak traffic times. In 15 min, only 2 and 8% of the population able to access health facilities had an accessibility index of ≥0.01 during peak and off-peak traffic times, respectively. This meant that at least one health facility per 100 people was within the catchment area. The rest had an accessibility index of <0.01, with one health facility having more than 100 people within the given threshold. In 30 min, the proportion of the population with an accessibility index of at least one heath facility per 100 population increased to 16 and 18% during peak and off-peak traffic times, respectively. In 45 min, two thirds of the population, 67% during peak time and 69% during off-peak hours had an accessibility index of one health facility offering primary health care to more than 100 people (<0.01) within the given threshold. The rest had at least one health facility per 100 people. On the other hand, <1% of the population, predominantly in the southwestern region, had a relatively higher accessibility index of at least two health facilities per 100 people within the three cut-off times, during peak and off-peak traffic periods (Figure 3).

**Figure 4:**
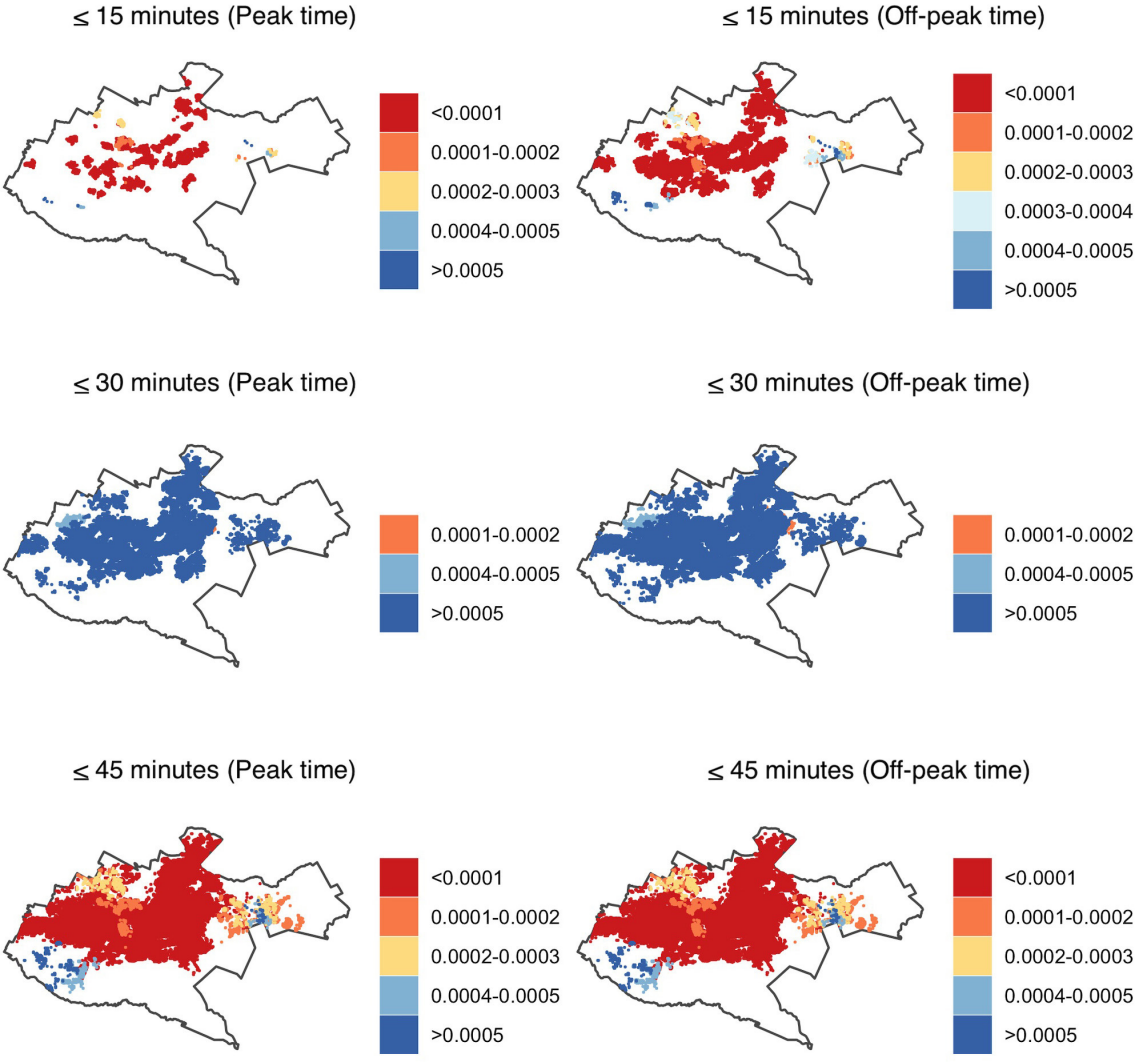
Accessibility index (population: facility ratios) for secondary healthcare in Nairobi within 15-, 30- and 45-min drive during peak and off-peak traffic times. The white areas have no accessibility to health facilities. Shapefile source: GADM (18).

In secondary health care facilities, the coverage was mainly in the central region, with the peripheries having a relatively better coverage when compared to primary health care facilities. In 15 min, the accessibility index for 99% of the population was lower than primary health care where more than 10,000 people had access to one hospital (<0.0001) both during peak and off-peak traffic times. In 30 min, majority of the population, 97% during peak and 96% during off peak traffic times had an accessibility index of at least five facilities per 10,000 people. In 45 min, 75 and 78% of the population had an accessibility index of <0.0001 where one hospital was primarily within access to more than 10,000 people during peak and off-peak traffic times respectively. Less than 1% of the population had a relatively better accessibility index of at least two facilities accessible to 10,000 individuals. The peripheries were underserved in accessing the health facilities in <30 min during peak and off-peak traffic scenarios as they had no access to a facility (Figure 4).

**Figure 5:**
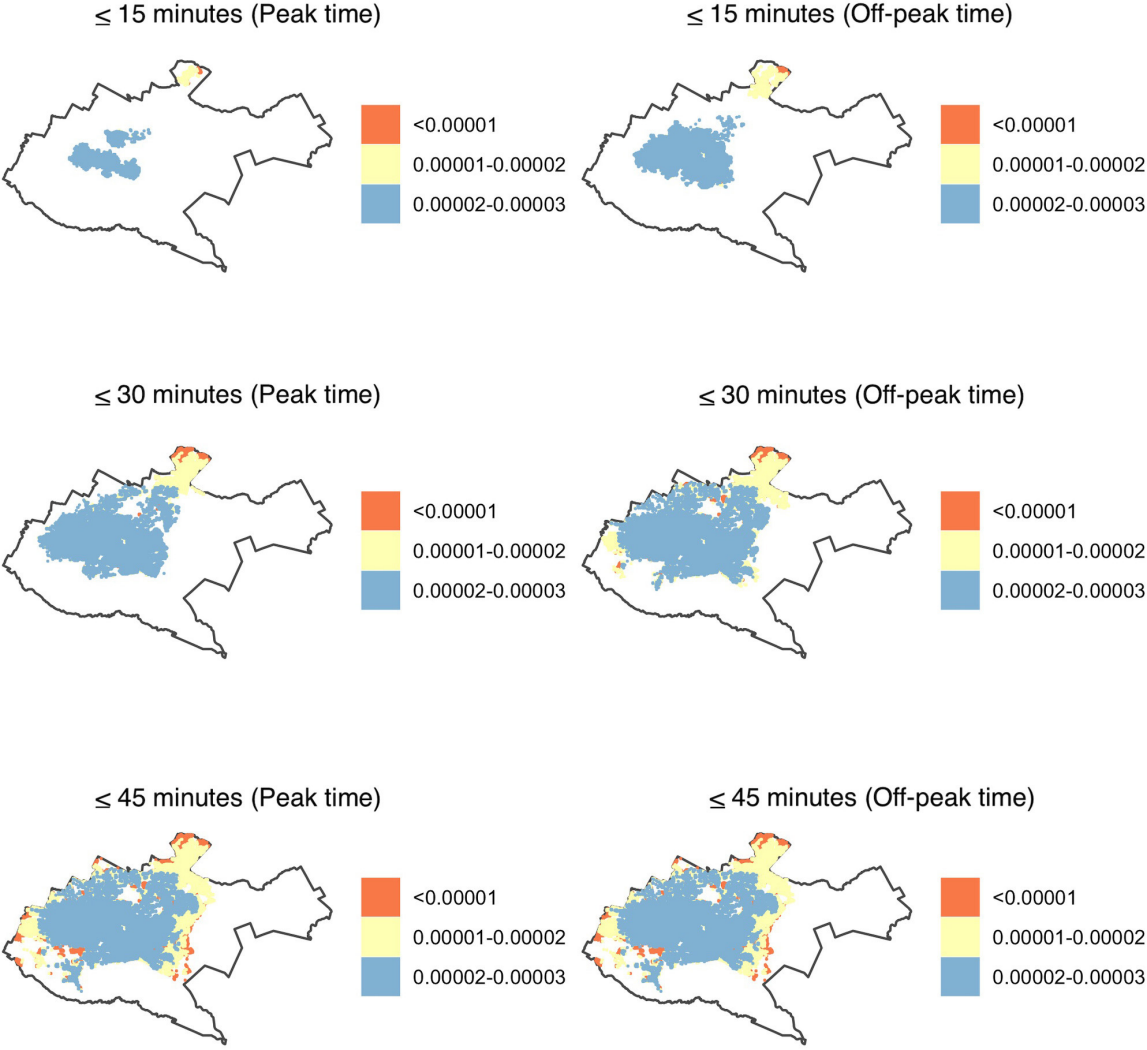
Accessibility index (population: facility ratios) for tertiary health care in Nairobi within 15-, 30- and 45-min drive during peak and off-peak traffic times. The white areas have no accessibility to health facilities. Shapefile source: GADM (18).

Similar to facilities offering primary and secondary health care, tertiary health facilities were mostly accessible to the population in the central region. The accessibility index of the number of health facilities to population ranged from 1 to 3 health facilities accessible to 100,000 people. In 45 min, 2 and 3% of the population had an accessibility index of more than 100,000 people to one facility whereas 80 and 86% of the population had a relatively better accessibility index of at least two facilities per 100,000 people during peak and off-peak traffic times, respectively. The eastern region and the peripheries were predominantly underserved with no secondary health facility within 15, 30, and 45 min drive time during peak and off-peak traffic scenarios (Figure 5).

### Ratio of the healthcare professionals to the population

All the population that was able to access health facilities offering tertiary care within 15 and 30 min had an optimal accessibility ratio of healthcare professionals which was above the recommended ratio of 4.45 per 1,000 people during peak and off-peak traffic scenarios. On the other hand, secondary health care facilities had the highest proportion of population that had a sub-optimal ratio of below 4.45 healthcare professionals per 1,000 people. In the proportion that was able to access secondary health care facilities in <30 min, more than 60% had a sub-optimal ratio of healthcare professionals during peak and off-peak traffic times. On the other hand, excluding population within 2.5 kilometer of the study area boundary significantly reduced the population with a sub-optimal ratio of healthcare professionals in both primary and secondary health facilities (Table 3).

**Table 3:** Population with a sub-optimal ratio of <4.45 healthcare professionals per 1,000 within 15, 30, and 45 min of primary, secondary, and tertiary healthcare in Nairobi, during peak and off-peak traffic times.

**Healthcare level**	**Time**	**Population in millions (%)**					
		<**4.45 healthcare workers per 1,000**			<**4.45 healthcare workers per 1,000**		
		**population (no buffer)**			**population (2.5 km buffer included)**		
		**Peak**	**Off-peak**	***P-*value**	**Peak**	**Off-peak**	***P*-value**
Primary	<15 min	0.2 (11%)	0.5 (25%)	<0.001	0.1 (9%)	0.3 (17%)	<0.001
	<30 min	1.0 (35%)	1.7 (55%)	<0.001	1.3 (33%)	1.5 (38%)	<0.001
	<45 min	2.5 (64%)	3.1 (78%)	<0.001	2.1 (53%)	2.6 (61%)	<0.001
Secondary	<15 min	0.8 (19%)	1.8 (44%)	<0.001	0.7 (17%)	1.6 (39%)	<0.001
	<30 min	2.5 (62%)	2.9 (71%)	<0.001	2.3 (58%)	2.6 (65%)	<0.001
	<45 min	4.1 (100%)	4.1 (100%)	1	3.2 (80%)	3.2 (80%)	1
Tertiary	<15 min	0	0	1	0	0	1
	<30 min	0	0	1	0	0	1
	<45 min	2.0 (49%)	2.7 (66%)	<0.001	1.4 (35%)	1.9(48%)	<0.001

**Figure 6:**
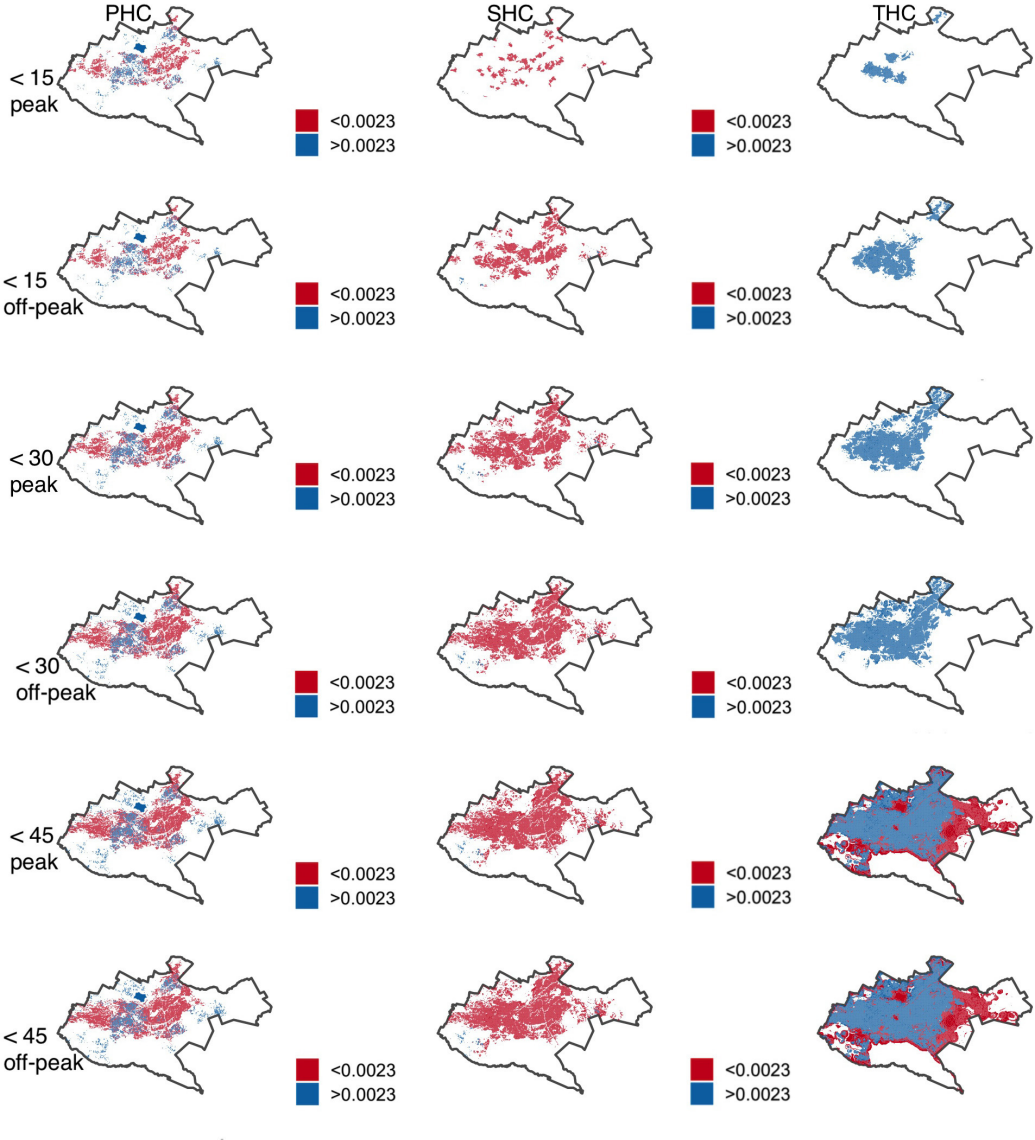
Accessibility index (population: health care professional ratios) by facility tier for primary (PHC), secondary (SHC) and tertiary (THC) health care in Nairobi within 15-, 30- and 45-min drive during peak and off-peak traffic times. The white areas have no accessibility to health care professionals. Shapefile source: GADM (18).

Spatial visualization of accessibility of healthcare professionals in Nairobi County varied, depending on journey time threshold, traffic conditions, and level of healthcare. Facilities offering tertiary healthcare were able to adequately cover the central and northern region of Nairobi, while primary health care facilities covered the western and eastern region. Secondary health facilities had a predominantly sub-optimal accessibility ratio in the central region (Figure 6). In <30 min, the population residing in the peripheries was mainly not able to access healthcare professionals, both during peak and off-peak traffic times.

## Discussion

In this study, we use the 2SFCA method to calculate the accessibility of the population to healthcare facilities and professionals, comparing peak and off-peak traffic times while accounting for edge effects. We report that traffic congestion plays a significant role on the proportion of population able to access the different levels of healthcare with more than a third of the population not able to access health facilities within 30 min drive time. Similarly, 4, 16, and 11% of the population can access facilities offering primary, secondary, and tertiary healthcare services during off-peak traffic times but experience poorer geographic access during peak traffic times. Patterns of inequality are observed where the peripheries are predominantly underserved by inaccessible health facilities and healthcare professionals during periods of traffic congestion. With the population able to access health facilities, a sub-optimal ratio of <4.45 healthcare professionals per 1,000 people is generally observed in facilities offering primary and secondary healthcare, with edge effects playing a significant role in the ratio.

These results are similar to those of two studies conducted in Dhaka and Nairobi which reported that traffic congestion had a significant impact on the physical accessibility of health facilities that offer emergency health care, with low accessibility indices of these health facilities observed in congested areas (15, 30). Transportation barriers to accessing amenities have been associated with delayed access in essential health services (34). A study in KwaZulu-Natal reported that the time spent by patients in commuting and at the health facility per month increased the likelihood of financial distress in a patient (35). This may be explained by the time spent away from the workplace, affecting the patients' income. To increase adherence to healthcare visits, one of the strategies would be to use traffic variation on physical access of health facilities to plan on the optimal placement of future healthcare facilities offering primary and curative services.

One of the primary goals of achieving universal coverage of essential health services for all by 2030 is to increase access to curative healthcare services and adequate workforce (4). However, results from our study showed sub-optimal access to healthcare professionals. Additionally, the WHO has reported sub-optimal availability of these professionals in developing countries, particularly in sub-Saharan Africa (36). This shortage may be explained by the multiple jobholding of healthcare professionals in developing countries who are mostly employed by government-owned health facilities, but also consult in privately owned hospitals, and also the emigration of healthcare professionals to developed countries (37, 38). The aging workforce and the imbalances in the distribution and skill mix composition of healthcare professionals in the different cadre and ownership status of health facilities has also contributed to the shortage in healthcare professionals (39).

A significant change in the ratio of healthcare professionals to population was observed during the implementation of edge effects, with the central regions enjoying relatively better access to health facilities and ratio of population to healthcare professionals. This was in contrast to results from a study in France that observed only minor variation in accessibility of healthcare when edge effects were considered (40). This difference in study findings may be explained by the structure of the city and the healthcare utilization of the population in the periphery in developing regions. Many LMICs have expanded rapidly outwards from a formally planned core, often with limited regulatory oversight or planning (41). This structure has influenced the peripheries as these areas are forgotten during planning and development of basic social services (42). One of the coping mechanisms of the peripheries has been utilization of healthcare services from neighboring areas which are relatively closer. This was observed in Ghana where residents in the peripheries utilized healthcare services from the neighboring areas (43). Peripheries should be included when planning the development of cities to ensure there is adequate service provided for all residents in the administrative boundary.

Similar to healthcare, a study conducted in Nairobi, a rapidly urbanizing city, reported that more than three quarters of the population in Nairobi was not able to access jobs in the city during peak time, with the population residing in peripheries being highly disadvantaged (44). These findings thus add to evidence that traffic variation plays a significant role in accessibility of services, and a multisectoral collaboration between the ministries of health and transport should be incorporated while optimizing placement of health facilities. The 2SFCA method should be used in the optimal placement of public amenities and services in congested cities. Specifically, this may be augmented with use of probe traffic data which is available from different commercial sources and with increasing global coverage in rapidly urbanizing LMIC cities (45).

One major assumption of this study, which has also been evidenced in previous studies, was the use of the 2SFCA as a key measure of spatial accessibility to healthcare services and professionals using equal accessibility of the population within the catchment area regardless of time taken to the health facility (46). Enhanced Two Step Floating Catchment Area (E2SFCA) and three-step Floating Catchment Area (3SFCA) methods have been proposed to account for this weakness, though a standard distance or time-decaying method is not yet proposed (47, 48). Another shortfall of the 2SFCA method was the unavailability of a standard metric on the minimum time that one ought to travel while seeking healthcare services and the ratio of healthcare professionals to population, making it difficult to compare study findings from different countries or populations (49). UHC, which seeks to ensure that every person should have equitable access to quality healthcare services which are available when needed, should propose a recommended time that should be taken in seeking the different levels of healthcare services. Similarly, a country specific recommended ratio of the cadre of healthcare professionals to population should be determined, while keeping in mind the distances to health facilities and the willingness-to-travel of populations in different settings in search of healthcare (47). Another assumption made in the study was the use of an average number of healthcare professionals to represent all the health facilities in a given service tier. The WHO has designed a registry of healthcare professionals with the aim of helping countries track their health workforce per facility (50). However, this data for Kenya was not readily available to allow comprehensive calculation of the accessibility index of the population to the healthcare workers (51). A future study should assess the number of healthcare professionals per health facility and determine the precise ratio between healthcare professionals and the population.

Routing services such as those provided by ESRI currently use historic traffic data to estimate mean journey times at specific times of week and day. From a health services planning perspective, unpredictability in travel times may give rise to missed appointments and healthcare system costs (52). Routing services that estimate the variance in travel times alongside the mean could thus support future studies of UHC in congested cities (34).

## Conclusions

We argue that traffic congestion should be incorporated by urban planners and policy makers while planning for healthcare and other services in rapidly urbanizing cities. Travel time taken to access a health facility has implications on delay and non-utilization of healthcare. Similarly, ratio of healthcare workforce to the population should be incorporated in planning for healthcare to optimize coverage during peak and off-peak traffic times.

## Data availability statement

The datasets presented in this study can be found in online repositories. The names of the repository/repositories and accession number(s) can be found below: https://osf.io/rjybc/OpenScienceFrameworks.

## Author contributions

NM, ST, and JW conceptualized the study. NM and MM analyzed the data. NM wrote the first draft of the manuscript. ST, JW, and HM offered mentorship and wrote the manuscript. All authors read and approved the final manuscript.

## Funding

The network routing analysis of the catchment of health facilities was funded by Africa Geoportal.

## Conflict of interest

The authors declare that the research was conducted in the absence of any commercial or financial relationships that could be construed as a potential conflict of interest.

## Publisher's note

All claims expressed in this article are solely those of the authors and do not necessarily represent those of their affiliated organizations, or those of the publisher, the editors and the reviewers. Any product that may be evaluated in this article, or claim that may be made by its manufacturer, is not guaranteed or endorsed by the publisher.

## Abbreviations

2SFCA: two step floating catchment area
ESRI: environmental systems research institute
GADM: global administrative area database
KSPA: Kenya service provision assessment
LMIC: low and middle income countries
PHC: primary healthcare
SHC: secondary healthcare
THC: tertiary healthcare
UHC: universal health coverage.
